# Scaling-up antiretroviral therapy in Malawi

**DOI:** 10.2471/BLT.15.166074

**Published:** 2016-07-27

**Authors:** Andreas Jahn, Anthony D Harries, Erik J Schouten, Edwin Libamba, Nathan Ford, Dermot Maher, Frank Chimbwandira

**Affiliations:** a HIV and AIDS Department, Ministry of Health, Lilongwe, Malawi.; b International Union against Tuberculosis and Lung Disease, 68 Boulevard Saint Michel, 75006 Paris, France.; c Management Sciences for Health, Lilongwe, Malawi.; d Independent public health consultant, Lilongwe, Malawi.; e Department of HIV and Hepatitis, World Health Organization, Geneva, Switzerland.; f Special Programme for Research and Training in Tropical Diseases, World Health Organization, Geneva, Switzerland.

## Abstract

**Problem:**

In Malawi, health-system constraints meant that only a fraction of people infected with human immunodeficiency virus (HIV) and in immediate need of antiretroviral treatment (ART) received treatment.

**Approach:**

In 2004, the Malawian Ministry of Health launched plans to scale-up ART nationwide, adhering to the principle of equity to ensure fair geographical access to therapy. A public health approach was used with standardized training and treatment and regular supervision and monitoring of the programme.

**Local setting:**

Before the scale-up, an estimated 930 000 people in Malawi were HIV-infected, with 170 000 in immediate need of ART. About 3000 patients were on ART in nine clinics.

**Relevant changes:**

By December 2015, cumulatively 872 567 patients had been started on ART from 716 clinics, following national treatment protocols and using the standard monitoring system.

**Lessons learnt:**

Strong national leadership allowed the ministry of health to implement a uniform system for scaling-up ART and provided benchmarks for implementation on the ground. New systems of training staff and accrediting health facilities enabled task-sharing and decentralization to peripheral health centres and a standardized approach to starting and monitoring ART. A system of quarterly supervision and monitoring, into which operational research was embedded, ensured stocks of drug supplies at facilities and adherence to national treatment guidelines.

## Résumé

**Problème:**

Au Malawi, en raison des contraintes du système de santé, seule une petite partie des personnes atteintes du virus de l'immunodéficience humaine (VIH) ayant besoin d'un traitement antirétroviral (TAR) immédiat pouvaient en bénéficier.

**Approche:**

En 2004, le ministère de la Santé a lancé des plans visant à élargir l'accès aux TAR dans tout le pays et à assurer l'accessibilité géographique équitable de ces traitements. La démarche s'est axée sur la santé publique, avec des formations et des traitements standardisés ainsi qu'une supervision et un contrôle réguliers du programme.

**Environnement local:**

Avant cette initiative, le Malawi comptait environ 930 000 personnes atteintes du VIH, dont 170 000 qui avaient besoin d'un TAR immédiat. Environ 3000 patients étaient sous TAR, dans neuf cliniques.

**Changements significatifs:**

En décembre 2015, au total, 872 567 patients avaient débuté un TAR dans 716 cliniques, suivant les protocoles nationaux de traitement et dans le cadre du système normalisé de suivi.

**Leçons tirées:**

La forte mobilisation nationale a permis au ministère de la Santé de mettre en place un système uniformisé afin d'élargir l'accès aux TAR et a fourni des points de référence pour sa mise en œuvre sur le terrain. Les nouveaux systèmes de formation du personnel et d'accréditation des établissements de santé ont permis de partager les tâches et de les décentraliser vers les centres de santé périphériques, tout en adoptant une démarche standardisée pour le commencement et le suivi des TAR. Les stocks de médicaments dans les établissements ainsi que le respect des directives nationales en matière de traitements ont été assurés grâce à un système de supervision et de contrôle trimestriels qui intégrait des aspects de recherche opérationnelle.

## Resumen

**Situación:**

Las restricciones del sistema sanitario en Malawi dieron lugar a que únicamente una fracción de las personas afectadas por el virus de la inmunodeficiencia humana (VIH) con necesidades inmediatas de obtener un tratamiento antirretroviral (TAR) recibiera tratamiento.

**Enfoque:**

En 2004, el Ministerio de Sanidad de Malawi lanzó planes para aumentar los TAR en todo el país, adhiriéndose al principio de equidad para garantizar el acceso geográfico justo a la terapia. Se utilizó un enfoque de salud pública con una formación y tratamiento estandarizados, así como la supervisión y control habituales del programa.

**Marco regional:**

Antes del aumento, había alrededor de 930 000 personas infectadas con VIH en Malawi, 170 000 de ellas con una necesidad inmediata de obtener TAR. Alrededor de 3 000 pacientes recibieron TAR en nueve clínicas.

**Cambios importantes:**

En diciembre de 2015, de forma acumulativa, 872 567 pacientes comenzaron con TAR en 716 clínicas, siguiendo los protocolos de tratamiento nacionales y utilizando el sistema de supervisión estándar.

**Lecciones aprendidas:**

Un fuerte liderazgo nacional permitió al ministerio de sanidad implementar un sistema uniforme para aumentar los TAR y ofreció referencias para su implementación en el terreno. Los nuevos sistemas de formación del personal y acreditación de instalaciones sanitarias permitieron el reparto de tareas y la descentralización a los centros sanitarios periféricos, así como un enfoque estandarizado para comenzar y supervisar los TAR. El sistema de supervisión y control trimestral, al que se incorporó una investigación operativa, garantizó reservas de medicamentos en las instalaciones y el cumplimiento de las directrices nacionales para el tratamiento.

## ملخص

**المشكلة:**

تمثلت المعوقات التي تواجه النظام الصحي في ملاوي في أن العلاج لم يصل سوى لنسبة ضئيلة فقط من المصابين بفيروس العوز المناعي البشري (HIV) ممن هم في أشد الحاجة إلى العلاج بمضادات الفيروسات القهقرية (ART).

**الأسلوب:**

أعلن وزير الصحة الملاوي في عام 2004 عن خطط لزيادة نسبة العلاج بمضادات الفيروسات القهقرية في جميع أنحاء البلاد، وذلك بغرض الالتزام بمبدأ المساواة لضمان الحصول على العلاج وتوزيعه بطريقة عادلة في كل مكان. وقد تم اتباع نهج الحفاظ على الصحة العامة من خلال تقديم التدريب والعلاج المتوافقين مع المعايير والإشراف والمراقبة المنتظمة على البرنامج.

**المواقع المحلية:**

قبل زيادة نسبة العلاج، كان عدد المصابين بفيروس العوز المناعي البشري في ملاوي يُقدّر بـ 930,000 شخص، مع وجود عدد يُقدّر بـ 170,000 شخص في أشد الحاجة لتلقي العلاج بمضادات الفيروسات القهقرية. وكان هناك ما يقرب من 3000 مريض يتلقون العلاج بمضادات الفيروسات القهقرية في تسع عيادات.

**التغيّرات ذات الصلة:**

بحلول شهر ديسمبر/كانون الأول من عام 2015، بدأ عدد إجمالي من المرضى يبلغ 872,567 مريضًا في تلقي العلاج بمضادات الفيروسات القهقرية من 716 عيادة، وذلك بالالتزام ببروتوكولات العلاج على المستوى الوطني واتباع نظام المراقبة القياسي.

**الدروس المستفادة:**

لقد أتاحت القيادة الوطنية القوية لوزارة الصحة فرصة تنفيذ نظام موحد لزيادة نسبة العلاج بمضادات الفيروسات القهقرية وتوفير المعايير المخصصة للتنفيذ على أرض الواقع. ولقد ساهمت الأنظمة الجديدة الخاصة بتدريب الموظفين واعتماد المرافق الصحية في تمكين تقاسم المهام واللامركزية بالنسبة للمراكز الصحية النائية واتباع نهج متوافق مع المعايير لبدء توفير العلاج بمضادات الفيروسات القهقرية ومراقبته. وقد تم فرض نظام ربع سنوي مخصص للإشراف والمراقبة – والذي تضمن البحوث الميدانية – والذي أدى إلى ضمان الحصول على مخزون إمدادات الأدوية في المرافق والتقيد بالمبادئ التوجيهية للعلاج على المستوى الوطني.

## 摘要

**问题:**

在马拉维，医疗体系的局限性意味着仅有一小部分感染艾滋病毒  (HIV)、急需抗逆转录病毒治疗的人得到了治疗。

**方法:**

2004 年，马拉维卫生部启动了多项计划以在全国范围内扩大抗逆转录病毒治疗的使用。计划遵循公平原则，以确保不同地域公平获得治疗。 使用了一套公共卫生方法，同时开展规范化培训与治疗以及定期项目监督与监控。

**当地状况:**

在扩大抗逆转录病毒治疗的使用之前，据估计，马拉维有 930 000 人感染了艾滋病毒，其中 170 000 人急需抗逆转录病毒治疗。大约 3000 名患者正在 9 家诊所接受抗逆转录病毒治疗。

**相关变化:**

截止到 2015 年 12 月，累计有 872 567 名患者已经按照国家治疗协议并使用标准监管系统在 716 家诊所开始接受抗逆转录病毒治疗。

**经验教训:**

强有力的国家领导允许卫生部实施统一的制度以扩大抗逆转录病毒治疗的使用，并为具体实施提供了基准。 通过员工培训和配备卫生设施的新制度，实现了任务共享并将任务分散到周边卫生中心，也为启动和监管抗逆转录病毒治疗提供了一套标准化方法。季度监督与管理制度包含操作研究，从而保证了库存药物在设施上的供应并且遵循国家治疗准则。

## Резюме

**Проблема:**

Вследствие различных препятствий, с которыми сталкивалась система здравоохранения в Малави, лишь часть людей, зараженных вирусом иммунодефицита человека (ВИЧ) и срочно нуждающихся в антиретровирусной терапии (АРТ), получала лечение.

**Подход:**

В 2004 году Министерство здравоохранения Малави приступило к выполнению планов по расширению охвата АРТ на территории всего государства, руководствуясь принципом равенства, чтобы обеспечить равномерный доступ к лечению для всего населения, независимо от географического положения. Была использована концепция общественного здравоохранения вместе со стандартизированным обучением и лечением, а также регулярным контролем и мониторингом программы.

**Местные условия:**

До расширения масштабов программы, согласно оценке, 930 000 жителей Малави были заражены ВИЧ и 170 000 из них срочно требовалась АРТ. Около 3000 пациентов проходили курс АРТ в девяти клиниках.

**Осуществленные перемены:**

К декабрю 2015 года в общей сложности 872 567 пациентов начали проходить АРТ в 716 клиниках в соответствии с национальными протоколами лечения и с использованием стандартизованной системы мониторинга.

**Выводы:**

Обладающее большими полномочиями политическое руководство страны позволило министерству здравоохранения внедрить единую систему для расширения АРТ и предоставило критерии прогресса в отношении практической реализации. Благодаря новым системам обучения персонала и аккредитации медицинских учреждений стало возможным разделение задач и рассредоточение функций между вспомогательными медико-санитарными центрами, а также применение стандартизованного подхода к началу АРТ и ее мониторингу. Система поквартального контроля и мониторинга, включающая оперативный анализ, гарантировала наличие запасов лекарственных средств в учреждениях и соблюдение национальных руководящих принципов лечения.

## Introduction

Malawi is a low-income country with an estimated population of 16 million in 2012.^1^ In 2004, approximately 930 000 Malawians were thought to be infected with human immunodeficiency virus (HIV), with an estimated 100 000 new HIV infections occurring annually and around 170 000 people in immediate need of antiretroviral therapy (ART).^2^ That year, the Malawian Ministry of Health decided to scale up ART nationwide.

Before the scale-up, only nine hospitals in the public sector were delivering ART to about 3000 patients and treatment was unstructured, few health-care workers had received formal training in ART and there were no national systems of monitoring, recording and reporting.^3^ This paper discusses the achievements of the scale-up in the face of severe health-system constraints and highlights the lessons learnt and new challenges.

## Public health approach

The health ministry assumed responsibility for the national scale-up of ART, adhering strongly to the principle of equitable access to therapy for everyone in Malawi, regardless of geographical location or type of health facility in the area. The implementing partners and stakeholders, including the private sector, worked together with the HIV department of the health ministry to develop national scale-up plans and implement one standardized system to deliver and monitor ART.

An effective system was already in place for diagnosing HIV infection. Criteria were developed for starting ART, based on the World Health Organization’s (WHO) clinical stage 3 and 4 disease, or a CD4+ T-lymphocyte count below 200 cells/μL. ART was to be delivered free of charge at all levels of the health service from central hospitals to local health centres, and the same methods were to be used for assessing patients for ART eligibility, initiating treatment, following-up patients, and monitoring, recording and reporting on treatment outcomes.

Plans for the initial and continued scale-up had clearly stated objectives and activities, with associated milestones and timelines, which provided a benchmark to assess implementation.^4^ The objectives included providing long-term ART, monitoring treatment outcomes on a quarterly basis, ensuring patients took at least 95% of their scheduled drugs, and having 50% of patients alive and on ART at three years from starting therapy. The activities included setting up the government HIV department, ensuring HIV testing, organizing procurement and distribution of drugs, and implementing supervision.

From the start, the Global Fund to Fight AIDS, Tuberculosis and Malaria financially supported the scale-up and remained the main funder. While having only one funding source meant less funding overall, it made it easier for the country to develop and implement a uniform approach that best fitted the country’s resources and infrastructure.

An intensive and innovative training schedule took place in the early months of 2004, focused on clinical officers and nurses learning the ART guidelines. The courses were country-wide, run by the HIV department and its partners, and focused on teaching health-care workers the ART guidelines, including how to monitor and do cohort reporting. All participants had to pass an examination with marks of at least 85%. Trained health workers were given checklists of activities to be undertaken when they returned to their facilities and were mandated to brief facility staff, the local government district headquarters and neighbouring health centres about ART. The HIV department carried out a formal structured accreditation of each ART facility before allowing health workers to start administering treatment. The accreditation included checking all the staff had been briefed, ensuring guidelines were in place, inspecting ART clinics and checking pharmacy security. The trainings were and continue to be done once for new sites, with shorter trainings repeated when new guidelines are developed and new treatment strategies are implemented and standardized.

To expand coverage, from 2004 onwards ART delivery was decentralized from hospitals to peripheral health centres, with responsibility for initiating treatment being devolved from doctors and clinical officers to also include medical assistants and nurses. This required dialogue and agreements with professional associations and regulatory bodies, after which guidelines about who could start patients on ART were included in subsequent editions of the national guidelines.

All ART facilities are responsible for quarterly reporting of cases and their treatment outcomes using ART patient treatment cards and patient registers. For facilities with a high burden of patients, a simple, real-time, touch-screen electronic medical record system has been introduced, with data entered by clinicians or nurses at the time of patient contact.^5^ The record system provides enhanced decision support to health-care workers during patient encounters, and the system produces quarterly and cumulative cohort reports.

Every three months, representatives from the HIV department and its partners conduct supervisory and monitoring visits to all ART sites in the country. This is to ensure there is adherence to guidelines, to check and collect data for national reporting and to monitor drug stock levels to facilitate drug supply forecasting.^6^ Operational research has been integrated into these supervisory visits, and country-wide data have been used to inform the programme in more detail about who is accessing ART and to provide answers to pertinent questions arising from field implementation and technical working groups.^3^

In the first few years of ART scale-up, the HIV department developed a system of drug forecasting and procurement. Facilities are graded according to the burden of disease in the community, based on HIV prevalence estimates, along with data on the number of new patients needing to start ART each quarter, the cumulative numbers of patients alive and ART drug stock levels.^7^ If facilities run short of drugs, the quarterly supervisory visits ensure that drugs can be moved from well stocked to understocked facilities.

## Achievements

Within six months of the launch of the scale-up plan, ART was being delivered at 19 health facilities within the public sector. In subsequent years, treatment was rapidly brought to scale in both the public and private sectors and by 31 December 2015, there were 716 ART clinics. Following national treatment protocols and using the standard monitoring system, public and private clinics had started a total of 872 567 new patients on ART, of whom 595 186 were alive and on ART by December 2015 (Table 1).^8^ At 64 government clinics throughout the country, 532 707 patients had been registered for ART through the electronic medical record system. During the first five years, from 2004 to 2008, representatives never found drugs to be out of stock, either nationally or at individual sites.

**Table 1:** Characteristics and outcomes of patients ever started on antiretroviral therapy in Malawi up to 31 December 2015

Characteristic	No. (%) of patients
**Total registered at ART clinics**	1 091 656 (100)
**Registration type^a^**	
First-time ART	872 567 (80)
ART re-initiation	12 334 (1)
ART transfer-in	206 755 (19)
**Sex**	
Male	393 498 (36)
Female	698 158 (64)
**Pregnancy status (*n* = 698 158)**	
Non-pregnant	564 661 (81)
Pregnant	133 497 (19)
**Age at ART initiation, years**	
≥ 15	997 286 (91)
0–14	94 370 (9)
**Reason for starting ART**	
Presumed severe HIV disease (infants)	3 707 (< 1)
Confirmed HIV infection^b^	
Stage 1 or 2	488 873 (45)
Stage 3	487 560 (45)
Stage 4	103 674 (10)
Other^c^	7 842 (< 1)
**Primary outcome (*n* = 888 918)^d^**	
Alive and on ART (includes patients in transit between sites)	595 186 (67)
Lost to follow-up	207 968 (23)
Stopped ART	4 293 (< 1)
Died	81 471 (9)
**Time of death, months after starting ART (*n* = 81 471)**	
1	19 542 (24)
2	12 275 (15)
3	7 393 (9)
4+	42 261 (52)

AIDS; acquired immunodeficiency syndrome; ART: antiretroviral therapy; HIV: human immunodeficiency virus; WHO: World Health Organization.

^a^ ART re-initiation refers to a patient who interrupted ART for 3 months or longer and then returned to treatment; ART transfer-in refers to a patient already on ART who transferred from one site to another.

^b^ WHO clinical stages: stage 1 or 2 (asymptomatic or with mild HIV-related symptoms and signs), stage 3 (severe HIV-related symptoms), stage 4 (AIDS-defining illnesses).

^c^ Includes patients started on ART for reasons outside of the guidelines and also patients for whom data are missing.

^d^ At 31 December 2015. The difference between the 1 091 656 clinic registrations and 888 918 outcomes is due to the more than 200 000 patients who transferred between sites and were therefore counted more than once.

Note: Inconsistencies arise in some values due to rounding.

## Lessons learnt and challenges

The key to rapid and massive scale-up in a country with shortages of health-care workers and weak laboratory infrastructure was standardization and simplicity (Box 1). The current national ART guidelines,^9^ first published in late 2003^4^ and updated every two to three years since, lay out the simplified and standardized approach for ART delivery in clinics in both the public and private sectors.

Box 1 **Summary of main lessons learnt**

- Strong national leadership allowed the health ministry to implement standardized systems for staff training, accrediting health facilities and delivering and monitoring antiretroviral therapy.
- A system of quarterly supervision and monitoring ensured that health facilities had stocks of drug supplies and adhered to national treatment guidelines.
- The strategy to treat all HIV-infected pregnant and breastfeeding women (option B+) means that Malawi is well placed to manage the World Health Organization’s recommended treat-all approach.

Challenges in the early stages of scale-up were the few children accessing ART, difficulties in managing patients with HIV-associated tuberculosis and high early death rates (i.e. in the first six months of ART). After identifying the causes, these issues have largely been overcome by changing to simpler ART regimens, educating communities about the need to get tested and assessed for ART at an earlier stage of immune suppression and ensuring all patients are started on both ART and co-trimoxazole at the same time.^10^ Published studies from other countries, combined with locally generated operational research, convinced the HIV department that adjunctive co-trimoxazole, administered over a patient’s lifetime before or at the time of ART, reduced early mortality.^11^ Policy and practice changes followed in 2007–2008 and co-trimoxazole is now routinely incorporated into the programme’s activities.

In 2010, Malawi was faced with implementing WHO’s new guidance on prevention of mother-to-child transmission of HIV. Decisions about how to manage HIV-infected pregnant women depended on timely and reliable CD4+ testing.^12^ With a limited capacity to achieve this, Malawi proposed a new strategy (option B+) to offer all HIV-infected pregnant and breastfeeding women lifelong ART regardless of WHO clinical stage or CD4+ count.^13^ Option B+ was introduced in July 2011^9^ and after the introduction, the large increase in women starting ART has brought challenges. These include large patient loads at antenatal clinics, the need to confirm all HIV-positive test results to minimize the risk of misdiagnosis, and retaining HIV-infected women on long-term treatment during breastfeeding and weaning.^14^ Solutions to the challenges include giving women three-monthly appointments, providing more support for confirming HIV-positive results, and better education of staff and patients about the need for lifelong ART even when patients are feeling well.

New challenges are arising. First, many patients are lost to follow-up (Table 1), regardless of their time on ART. This may be partly due to poorer skills among the staff in peripheral facilities and omitting to notify when patients transfer between treatment sites. Second, a smaller percentage of patients (1%) are on second-line ART than may be expected (15–25%),^15^ raising concerns about the failure to identify patients who might have failed first-line therapy. Third, the burden of quarterly country-wide supervision for the large number of sites (over 750) is growing, mainly due to the need for transport to the sites, for skilled supervisors, and for funding. Fourth, there is the problem of ensuring regular drug stocks now that more ART regimens and larger quantities of drugs are needed compared with 10 years ago. Fifth, there is the issue of recognizing and managing new threats to health for treated patients, such as from noncommunicable diseases that are emerging due to HIV and ART or from patients’ ageing now that survival is prolonged.^16^

In 2014, the Joint United Nations Programme on HIV/AIDS declared the 90–90–90 treatment target to diagnose 90% of all people with HIV, provide antiretroviral therapy for 90% of those diagnosed and achieve viral suppression for 90% of those on treatment, by 2020. WHO guidance in 2015 recommended that ART be initiated in everyone living with HIV at any CD4+ count.^17^ Malawi’s implementation of option B+ makes the country well placed to embrace and manage WHO’s treat-all approach.
